# Submucosal tunneling endoscopic resection for the treatment of infantile gastric duplication cyst: the first report in infants

**DOI:** 10.1055/a-2686-2862

**Published:** 2025-10-07

**Authors:** Hanhua Zhang, Huanyu Liu, Xiaoxia Ren, Kuku Ge, Pinghong Zhou, Ying Fang

**Affiliations:** 1 Department of Gastroenterology, Xi’an Children’s Hospital and National Regional Medical Center for Children (Northwest), Shaanxi, China; 2 92323 Endoscopy Center and Endoscopy Research Institute, Zhongshan Hospital, Fudan University, Shanghai, China

A 5-month-old boy, diagnosed with gastric duplication by fetal magnetic resonance examination and occasional vomiting after birth. Abdominal ultrasonography showed a cystic mass in the left upper abdomen with gastric duplication. Abdominal CT examination: cystic mass at the esophagogastric junction, esophageal/gastric duplication ( Fig. 1 **a** ). Submucosal tunneling endoscopic resection (STER) was performed ( Video 1 ). During the procedure, an extragastric cyst at the esophagogastric junction, diagnosed as a gastric duplication malformation ( Fig. 1 **b, c** ). At 16 cm from the incisors, a Helabot knife performed submucosal injection and mucosal incision on the esophageal posterior wall ( Fig. 1 **d** ). A submucosal tunnel was established toward the cardiac lesion, revealing a cystic protrusion. Using an IT knife, circumferential dissection was performed, followed by en bloc resection with a disposable snare ( Fig. 1 **e, f** ). Minor bleeding at the resection site and tunnel was controlled by electrocoagulation at seven points using a thermal biopsy forceps. The tunnel entrance was closed with seven disposable titanium clips ( Fig. 1 **g, h** ). The pathological diagnosis was gastric duplication ( Fig. 1 **i** ). After 20 months of follow-up, the patient had no special discomfort and developed well.

Submucosal tunneling endoscopic resection for treatment of infantile gastric duplication cyst.Video 1

**Fig. 1:**
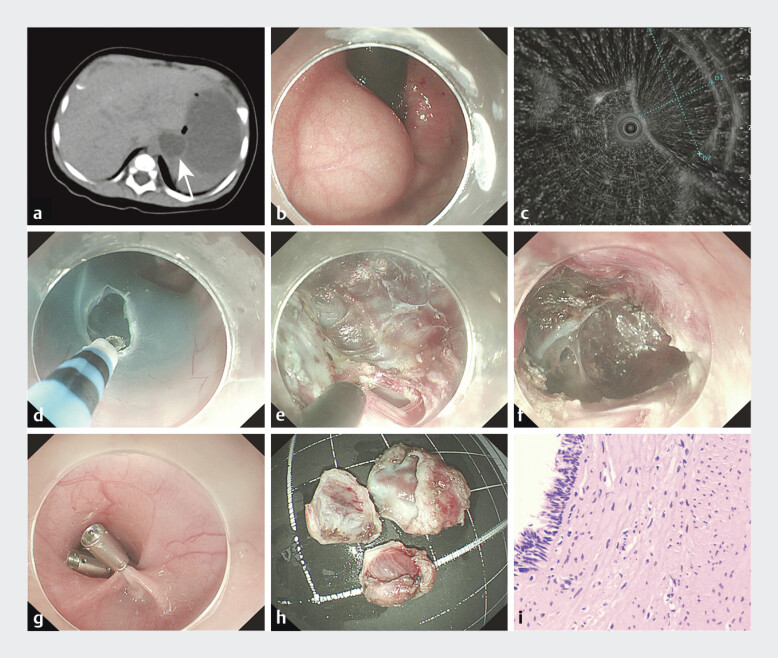
Submucosal tunneling endoscopic resection for treatment of infantile gastric duplication cyst. **a** Abdominal CT before STER, gastric repetitive malformation cyst was found at the esophagogastric junction (white arrow). **b** Gastroscopy revealed a submucosal tumor at the esophagogastric junction. **c** Endoscopic ultrasound showed a submucosal cyst. **d** Submucosal incision. **e** The cyst was cut open after the tunnel was completed. **f** Complete excision of cyst. **g** Closed tunnel opening. **h** A segmented cyst. **i** Gastric duplication was diagnosed by pathology.

Gastric duplication in children is a rare malformation of the digestive tract, with an incidence of about 17 per 1 million 1 ; the only option is surgical resection. Ye et al. reported a case of a gastric duplication cyst associated with an ectopic pancreas that was successfully resected with ESD 2 . The STER technology enhances the resection of lesions by creating a submucosal space prior to the procedure, thereby preserving the mucosal layer. This technique leverages the bodyʼs natural cavities to optimize surgical outcomes. STER represents an innovative surgical approach for the treatment of gastric duplication malformations and warrants consideration for integration into clinical practice.

Endoscopy_UCTN_Code_TTT_1AO_2AG_3AZ

## Notes

Endoscopy E-Videos https://eref.thieme.de/e-videos
*E-Videos* is an open access online section of the journal *Endoscopy* , reporting on interesting cases and new techniques in gastroenterological endoscopy. All papers include a high-quality video and are published with a Creative Commons CC-BY license. Endoscopy E-Videos qualify for HINARI discounts and waivers and eligibility is automatically checked during the submission process. We grant 100% waivers to articles whose corresponding authors are based in Group A countries and 50% waivers to those who are based in Group B countries as classified by Research4Life (see: https://www.research4life.org/access/eligibility/ ). This section has its own submission website at https://mc.manuscriptcentral.com/e-videos .
